# Better richer than environmentally friendly? Describing preferences toward and factors affecting precision agriculture adoption in Italy

**DOI:** 10.1186/s40100-023-00247-w

**Published:** 2023-05-31

**Authors:** Stefania Troiano, Matteo Carzedda, Francesco Marangon

**Affiliations:** 1 Department of Economics and Statistics, University of Udine, Udine, Italy; 2 Department of Economics, Business, Mathematics and Statistics, University of Trieste, Trieste, Italy

**Keywords:** Precision agriculture, Sustainability, Preferences

## Abstract

Precision agriculture is expected to support and strengthen the sustainability of food production. In spite of the demonstrated benefits of the application of Information Technology to improve agricultural practices, such as yield increase and input reduction, in Italy its adoption still lags behind. In order to understand limits of and perspectives on the adoption of such technologies, we conducted an explorative study. A survey with a choice experiment was carried out in Italy among 471 farmers and people interested in agricultural machinery and technologies. The results highlight how specific factors, such as excessive costs and lack of incentive policies, may limit the spread of precision agriculture. Conversely, the provision of adequate technical support would likely favor its adoption. Furthermore, latent class modeling was used to identify three segments of potential buyers: sustainability seekers; precision agriculture best features supporters; low emissions fans. Potential policy and market implications of this explorative study are discussed in the conclusion.

## Introduction

Precision agriculture (PA) is likely to play a relevant role in the transition toward sustainable agriculture (Balafoutis et al. 2017; Lee et al. 2021). PA utilizes Information Technology (IT), satellite technology, Geographical Information System (GIS) and remote sensing to improve agricultural functions and impacts (Belcore et al. 2021). It relies upon mobile apps, smart sensors, drones, cloud computing, artificial intelligence, Internet of Things (IoT) and blockchain to manage both environmental and socio-economic effects related to agricultural activities (Torky and Hassanein 2020; Zhao et al. 2021).

Besides providing material support to production processes, PA aims at enhancing agricultural efficiency (Romanelli et al. 2022; Schimmelpfennig 2016) and reducing its environmental impacts (Cisternas et al. 2020; Loures et al., 2020; Medici et al. 2019, 2021; Pierpaoli et al. 2013). In the past years, with the increase in cultivated areas and the advent of agricultural mechanization, heavy and widespread use of fertilizers has taken over together with conventional agriculture, ignoring the optimization of their application over time and space. By minimizing required inputs (water resources, fertilizers, plant protection products, etc.), improving quality of production and increasing yield quantity, PA helps enhancing agricultural socio-economic sustainability (Knoll et al. 2018; Patrício and Rieder 2018). Furthermore, it contributes to reduce the negative environmental impacts of agriculture by using fewer and more controlled resources to achieve the same or even better results (Mogili and Deepak 2018).

PA technologies are able to provide accurate diagnosis and management support to agricultural entrepreneurs, both in terms of yield productivity and profitability, including the controlled use of inputs with social, economic, and environmental—primarily climatic, benefits (Blasch et al. 2022). PA has been pioneered as a management tool in the grains industry (Whelan and Taylor 2013).

Moreover, in the past years, according to Colussi et al (2022), because of the COVID-19 pandemic, the increasing digitalization of the agricultural sector has become even more indispensable.

Nevertheless, in spite of the advantages that these technologies are expected to generate, PA adoption is still slow and lagging (Bucci et al. 2018; Finco et al. 2021). In particular, according to Lowenberg-DeBoer and Erickson (2019), medium and small farms in the developing world fall behind, as access to agricultural mechanization is less widespread.

Extensive understanding of the factors affecting adoption of this equipment is needed to adequately inform and support the development of PA technology and the programs diffused to promote its adoption. A number of studies tried to analyze this aspect (Pallottino et al. 2018) and several models were proposed to explain PA technology adoption (Carli et al. 2017). Aubert et al. (2012) tested a model to explain limits and barriers to PA technology adoption: the results highlight the importance of compatibility among PA technology components as well as the crucial role of farmers' expertise. Furthermore, according to Pierpaoli et al. (2013), PA adoption is mostly influenced by availability of financial resources, farmers’ socio-demographic characteristics (as for example education and computer literacy) and competitive and contingent factors (e.g., farm size, soil and landscape characteristics, geographical location). A broader approach characterizes the conceptual model proposed by Monteleone et al. (2019), who analyze the role of different actors operating in the farm and technology ecosystem, and respective actions and strategies that can be implemented to widen PA adoption. More recently, Vecchio et al. (2020) underlined the fundamental role of context-related factors to be explored in order to specify uptake of PA technologies. Both Liu et al. (2021) and Pathak et al. (2019) published the literature reviews they undertook with the aim to explore the processes of adoption of PA technologies. They found that very few studies examined multiple components of the complex adoption process. Researches were mainly focused on assessing the impact of a single determinant while neglecting the complexity and the multidimensional nature of the adoption process, which was consequently poorly represented (Giua et al. 2022). Recently, for example, Kleftodimos et al. (2022) studied the tools that can be used to convince farmers to adopt PA practices. More specifically, they analyzed Greek farmers’ PA adoption decisions in dairy production systems using a bio-economic model based on mathematical programming methods. Their results highlighted that the adoption of PA practices led to significantly better economic and environmental outcomes, and different levels of incentives can be efficiently targeted to encourage the adoption. Giua et al. (2022) used Structural Equation Modeling (SEM) and a Zero-Inflated Poisson Regression (ZIP) to investigate the intention to use and the actual adoption of PA, stating that farmers’ intention mainly relies on performance expectancy and complexity of technologies, as well as farmers’ social and peer pressure. Tey and Brindal’s (2021) meta-analysis found that perceived profitability, consultants and computer literacy have a moderate effect in driving the adoption of PA. Applying pairwise best–worst choice experiments, Thompson et al. (2019), used data from a phone survey on U.S. commercial crop producers to identify farmers’ perceptions over the benefits provided by PA technologies, which are considered as the main reasons behind adoption decisions.

To our best knowledge, only few studies have adopted the choice experiment methodology to analyze farmers’ preferences toward PA comparing both socio-economic and environmental aspects (e.g., Blasch et al. 2022). To fulfill this gap and provide insights on the determinants and barriers to the adoption of these technologies in Italy, a survey was carried out to better understand purchase choice. More precisely, given the lack of a clear distinction in the literature between the “adoption” of innovations as a multidimensional and multiphase process (i.e., “awareness”, “interest”, “evaluation”, “trial”, “adoption” according to the Diffusion Theory) (Rogers 1962, 1995), and the concept of adoption as a purchase choice, our study focuses on this second aspect, identifying both preferences and willingness to pay (WTP) of end users. Rather than exploring the factors which influence the whole adoption process, our exploratory study tried to describe socio-economic characteristics, habits and behavior of potential adopters. To reach this objective, a survey and a choice experiment (CE) were administered to Italian farmers and people interested in agricultural machinery and technologies, both to identify the effect of each considered characteristic on respondents’ choice and to highlight the presence of heterogeneity among respondents’ preferences.

The paper is organized as follows: Introduction provides a short overview of the theoretical background; Sect. "Materials and methods" describes the materials and methods used; Sect. "Results and discussion" presents the results and discussion; finally, concluding remarks are discussed in the conclusion.

## Materials and methods

To achieve the aforementioned aim, a survey was carried out through both face-to-face interviews to attendees at the International Exhibition of Agricultural and Gardening Machinery (EIMA International) in Bologna (IT) in 2018 and on-line questionnaires spread through specific agri-sector webpages. The web survey was launched on November 20th, 2018 and closed on April 6th, 2019. Participants were recruited via advertisements on two platforms (Google and Facebook). The face-to-face survey was also fielded from November 6th to November 11th, 2018 and it was preceded by a three-hours training session attended by an internal research interviewer. As is usual in this kind of surveys, interviewees were recruited among EIMA International participants. A random sampling approach was adopted according to Rossi et al. (2013).

In this paper, the questionnaire intended to analyze farmers and other agricultural industry operators’ knowledge of PA and preferences toward its adoption. Of the two sections of the questionnaire, the first one investigated knowledge over, and effective use of PA in the Italian agricultural industry, while the second one included a CE to collect and then understand potential customers’ preferences toward characteristics of a hypothetical PA technology investment. The CE is a well-known social science approach, widely adopted in existing studies to observe respondents' selection within a setting context to determine their utility and then analyze WTP (Batsell and Louviere 1991; Hensher 1994; Louviere 1991; Louviere et al 2000). In a CE, participants are presented with hypothetical but realistic choice situations. CE application derives from a solid theoretical background since it is based on both Lancastrian consumer theory (Lancaster, 1966) and random utility models (Block 1974; McFadden 1976), which are combined with some distinguishing features. According to Lancaster (1966), CE assumes that utility is derived from properties (i.e., characteristics) of a good rather than directly from it. Consequently, utility becomes a function of good characteristics and random utility models are applied to take into account the unobservable or random part of utility. In CE, the valued good is split into its key attributes, firstly allowing the trade-offs to be estimated between them. Secondly, CE can be applied to both ex-ante and ex-post valuations where good characteristics not yet in existence can be presented to respondents using a number of hypothetical scenarios.

For this reason, under the rationality assumption, a PA technology is chosen if the utility derived from it is greater than that derived from another good present as an alternative in the available choice set.

Two focus groups were conducted at the end of summer (late August) 2018 in a single round with a number of farmers and other stakeholders in order to identify the PA technologies to be analyzed, their level of adoption, their attributes and discuss questions in the questionnaire. Based on the focus group discussions, five attributes were identified, which were considered to be important for purchase decision, to compare alternative PA technologies (Table 1).

**Table 1:** Attributes and their corresponding levels

Attribute	Levels
Yield increase	Significant; moderate; low
Fuelling	Electric; hybrid
Brand	Industry leader; small firm
Greenhouse gas emissions	High; moderate; low
Price (€/PA equipment)	17,000: 30,000; 50,000

As detailed above, in a CE the attributes of a good/service are its characteristics, while their levels refer to the possible different configurations of each attribute. To make the attributes and their levels more understandable to the respondents, a hypothetical “PA equipment” was described to respondents as a tractor equipped with both automated guidance and autosteer, and data technologies such as yield monitor, precision soil sampling, and variable rate fertilizer application (Griffin et al 2018). According to a number of studies (Erickson et al. 2018; Fausti et al. 2018; Kitchen et al. 2002; Mitchell et al. 2018), this kind of PA equipment was widely analyzed among scholars to discuss specific barriers to adoption of this technology and better understand how to improve the agricultural workforce skills in using it.

The first attribute we considered was the potential increase in yields, which seems to be a preferred characteristic of farmers across all country (Blasch et al. 2022; Pedersen and Lind 2017; Silva et al. 2011). The yield increase attribute presented three levels of study: significant, moderate and low.

The second attribute offered respondents the opportunity to choose between electric and hybrid equipment. Gonzalez-de-Soto et al. (2016,2018) and Li et al. (2020) demonstrated the importance of hybrid energy systems in PA to obtain significant reductions in the emissions of atmospheric pollutants when discussing about and predicting PA adoption.

With regard to reputation and size of the precision technology provider, the third attribute, we proposed two alternative levels, namely: industry leader or small firm brand. This attribute gave respondents the opportunity to compare goods supplied by leading providers of precision agriculture and small firms’ provision. According to Mitchell et al. (2012), marketing activities of owner managers within small and medium enterprises (SMEs) are different, in that SME firms are not just small versions of their larger counterparts. In addition, small firms are more likely to have lower brand recognition and be a riskier purchase proposition for the buyer (Vaidyanathan and Aggarwal 2002).

Consumers’ increasing awareness and concern over negative impacts of agri-food activities has led to a need for differentiation in production methods. Consequently, we considered three different levels of greenhouse gas emissions: high, moderate, low.

The price attribute presented the levels € 17,000, € 30,000 and € 50,000, which were established according to EIMA decision makers estimates emerged in focus groups’ discussion. Each price level corresponded to a complete PA setup.

To efficiently elicit respondent preferences for the attributes, a fractional factorial orthogonal design was used to vary all attributes among the scenarios. Interviewers had to face six choice sets with three treatment combinations each, plus the opt-out alternative (“None of these”), which was present for those respondents who were not interested, to ensure that this survey was as realistic and practical as an investment opportunity in the real world.

**Fig. 1:**
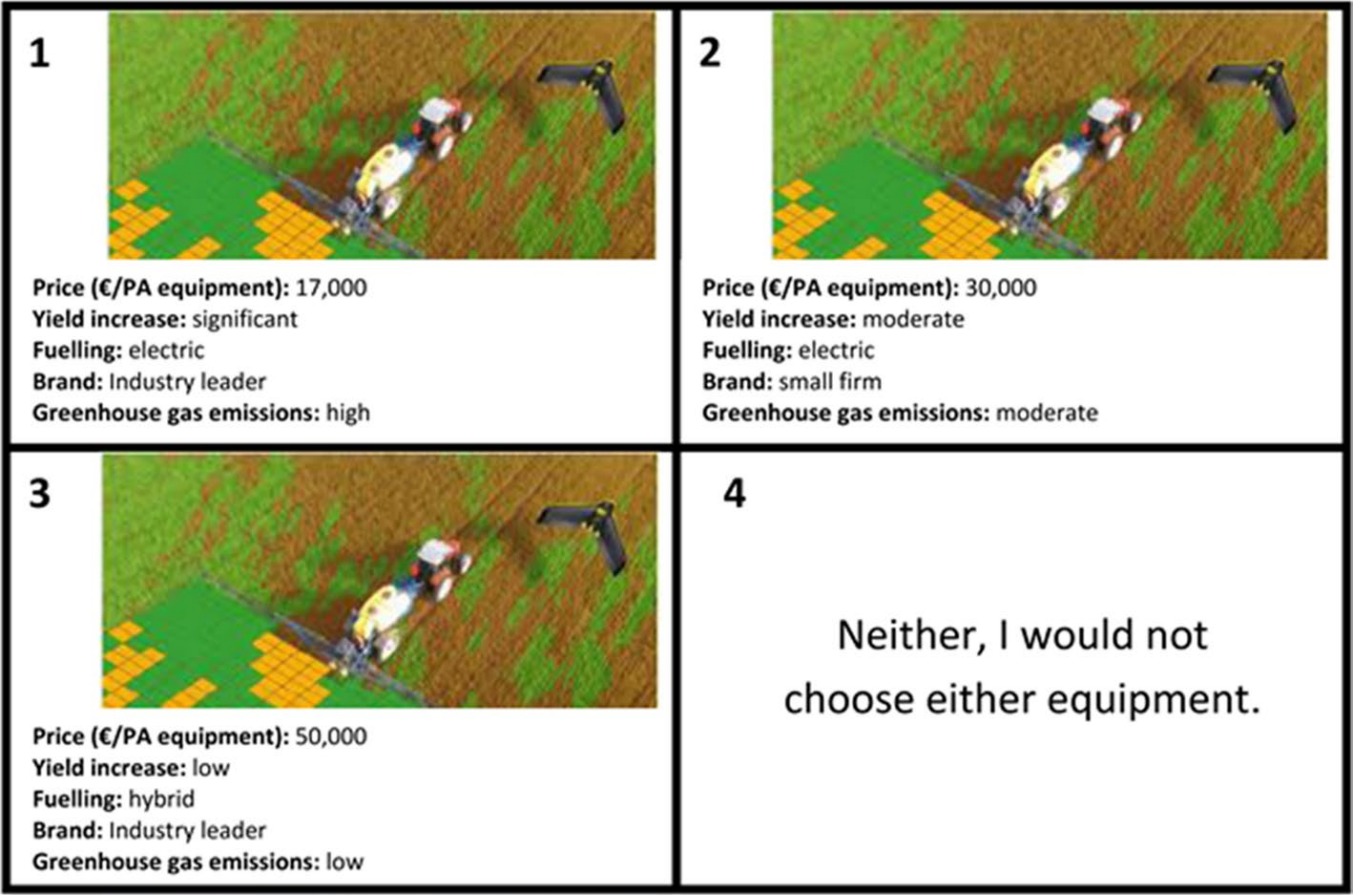
A choice set

The choice sets were shown in color pictures to the respondents. In detail, the respondents were asked to choose among three alternative PA systems. An example choice set is represented in Fig. 1. Furthermore, to let respondents better understand the presented PA equipment and improve the accuracy of their answers, the concept of PA was described with color pictures and wording at the beginning of CE. To avoid misunderstanding attributes and their levels were briefly explained to respondents (Johnston et al. 2017). However, since in the time of focus groups discussion links between PA adoption, yield increases and environmental improvements, including greenhouse gas emissions were not quantified (Schimmelpfennig 2018), interviewees were told that a “significant” yield increase corresponded to “more than 5%”, a “moderate” from 2 to 5% and “low” less than 2%. All these values were adopted on the basis of focus groups discussion and a literature review. Similarly, considering also that PA technologies vary widely in their contributions to effecting reductions in greenhouse gas emissions (Soto Embodas et al. 2019) and according to previous studies (e.g., Perez Dominguezet al. 2016), respondents were told that “high” greenhouse gas emission corresponded to PA technology able to reduce less than 30% of conventional technologies emissions, “moderate” from 30 to 50% emissions reduction, and “low” more than 50%.

About 30 pre-tests of the questionnaire were made before its actual administration. These pre-tests resulted in minor revisions in the formulation of questions.

**Fig. 2:**
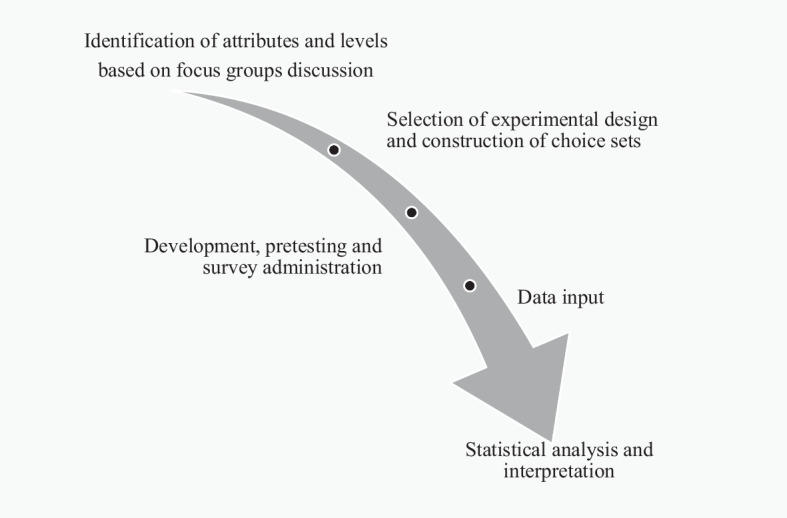
Key research stages of the choice experiment

Figure 2 points out the main stages we developed in conducting the CE according to our research objectives (Hanley et al. 1998).

## Results and discussion

Data collection took place between November 2018 and April 2019 among different age categories, and 471 total responses were obtained. In detail, 205 questionnaires (43.52%) were collected in person and 56.48% (266 respondents) on-line. Clearly, face-to-face questionnaires collected at EIMA may be biased, as participants would presumably be more interested in PA. Given the exploratory purpose of this study, this was deemed acceptable with the awareness that our findings are not generalizable.

Most survey participants were males (407 respondents). This result seems to be reasonable considering that the vast majority of farmers are male (Istat 2022). Furthermore, the average interviewee was 33 years old, lived in Northern Italy (70.91%), had completed high school (56.05%) (Table 2), and was mainly self-employed (61.57%). Table 2 and Table 3 provide further information on respondents’ education and employment condition. As stated above, respondents’ higher interest in PA may limit sample representativeness of the general population working in the agricultural industry.

**Table 2:** Respondents’ educational level

Educational level	Percentage (number of respondents)
Primary school	0.21% (1)
Secondary school	13.80% (65)
Some years of high school	9.13% (43)
High school	56.05% (264)
Degree	19.11% (90)
Other	1.70% (8)

**Table 3:** Professional position among agribusiness

Professional position in the agribusiness	Percentage (number of respondents)
Farmers	61.57% (290)
Consultants	6.79% (32)
Retailers	2.76% (13)
Machineries producers	2.55% (12)
Other roles	26.33% (124)

In detail, 290 respondents (i.e., the abovementioned percentage of interviewers who declared to be self-employed, 61.57%) were farmers, while the others were involved in different roles and positions in the agribusiness industry.

Respondents’ PA knowledge level was analyzed. As expected, findings confirm the limited adoption of PA in Italian farms, with only 116 respondent (35.91%) being users or owners of PA technology. Automatic driving devices (e.g., precision automatic steering motor for agricultural machineries, automatic steering wheel motor, electric steering motor torque) are the most widespread PA technologies among respondent farms (82 interviewees), while only one respondent declaring to use all the presented PA tools (i.e., automatic driving, interconnected machines, field data collection, mapping and data management, remote sensing, sensors and isobus).

More than half (62.75%) of the sample identified excessive costs as the main barrier to PA technology adoption. In addition, they declared they would purchase such technologies if public incentives and adequate technical support and assistance from resellers would be available. These findings could be linked to the structural characteristics of farms (i.e., size). Even though, due to limited resources devoted to this exploratory study, farm structure was not considered, the literature largely confirms the role of size in explaining the degree of digitalization (e.g., Ali 2012; Jorge-Vázquez et al. 2021).

Furthermore, respondents were asked to state their degree of agreement with and importance of a set of statements on various aspects of PA, using a Likert scale ranging from 1 (i.e., not very important/I do not agree) to 5 (i.e., very important/I very much agree). The replies showed the existence of widespread sensibility and awareness of the opportunity to minimize environmental damage, increase quality of agricultural products, allow collection of useful data, support decision making processes, reduce production costs and increase income. Table 4 further describes these aspects. In detail, respondents were mainly aware about the importance offered by PA to allow the collection of useful data: 85.13% pointed out this opportunity as important and very important. Then, 80.26% identified PA as much and very much able to increase income, while a lower percentage of respondents (67.94%) were much and very much convinced that PA supports decision making processes. 64.97% of respondents much and very much agreed about the opportunity offered by PA adoption to minimize environmental damages and 64.12% described PA as much and very much able to reduce production cost. Lastly, 56.47% were much and very much convinced about the capability of PA to increase the quality of agricultural products.

**Table 4:** Respondents’ knowledge and awareness about PA—Percentage

Statements about PA:	1 = not very important/I do not agree (%)	2 (%)	3 (%)	4 (%)	5 = very important/I very much agree (%)
Minimizes environmental damages	4.03	7.43	23.57	33.76	31.21
Iincreases quality of agricultural products	3.71	9.77	30.15	34.39	22.08
Allows collection of useful data	0.64	2.76	11.46	26.96	58.17
Supports decision making processes	4.46	7.01	20.59	31.21	36.73
Reduces production costs	1.91	4.25	29.72	33.12	31.00
Increases income	1.06	2.55	16.14	40.98	39.28

Finally, the CE was carried out to better identify purchasing preferences of potential buyers in the agricultural sector toward the previously described PA equipment setup. Preference elicitation allows estimation of the relative importance of different characteristics of PA machinery, trade-offs between these aspects and the total satisfaction or utility that responders derive from this technology. Choice responses (number of observations = 2826) were initially modeled using multinomial logit (MNL) (McFadden 1974) (Table 5), even though a latent class (LC) model was also tested to allow for unobserved heterogeneity in preferences (Boxall and Adamowicz 2002; Greene and Hensher 2003; Pacifico 2012; Train 2008). While MNL allows simple preference assessment modeling, the Independence of Irrelevant Alternatives (IIA) and uncorrelated unobserved error over time assumptions determine inability to account for heterogeneity, as preferences are considered fixed among all respondents. Using Mixed logit and LC models, on the contrary, we can relax the IIA assumption and account for preference heterogeneity.

**Table 5:** Multinomial logit and latent class models

Variable	MNL	Latent Class Model					
		Class 1		Class 2		Class 3	
	Coeff. (S.E.)	Coeff. (S.E.)	WTP° (€000/equipment)	Coeff. (S.E.)	WTP (€000/equipment)	Coeff. (S.E.)	WTP (€000/equipment)
OptOut	-1.23 (0.13)***	-2.30 (0.18)***	–	3.32 (0.47)***	–	-5.65 (1.23)***	–
Price	-0.03 (0.00)***	-0.01 (0.00)***	–	-0.02 (0.01)***	–	-0.19 (0.01)***	–
Significant yield increase	0.16 (0.06)***	0.03 (0.07)	–	1.07 (0.21)***	62.96	-2.12 (1.02)***	-11.40
Low yield increase	-0.49 (0.10)***	-0.96 (0.12)***	-68.94	-1.07 (0.37)***	-63.10	-2.13 (1.06)***	-11.46
Electric	0.09 (0.09)	0.31 (0.11)***	22.11	1.36 (0.33)***	80.34	-3.77 (1.18)***	-20.24
Industry leader	0.26 (0.11)***	0.11 (0.13)	–	1.60 (0.39)***	94.28	3.36 (2.04)	–
Low emissions	0.22 (0.06)***	0.22 (0.08)***	15.96	0.45 (0.27)**	26.47	2.04 (0.32)***	10.98
High emissions	-0.82 (0.11)***	-0.94 (0.14)***	-67.84	-1.89 (0.51)***	-111.77	0.36 (1.12)	–
Average probability	0.63		0.16		0.21		

***Significant at a 95% conf. level; ** Significant at a 90% conf. level

°Willingness to Pay is expressed in thousands € / equipment

By means of the program NLogit6®, both base and LC models were estimated. These models shared the same linear utility function, which is illustrated as follows:

$$\begin{aligned} {\text{U}}\left( {{\text{xi}}} \right) \, & = {\text{ Opt}} - {\text{Out}} + \, \beta_{{1}} \cdot {\text{SignificantIncrease}}_{{\text{i}}} + \, \beta_{{2}} \cdot {\text{LowIncrease}}_{{\text{i}}} \\ & \quad + \, \beta_{{3}} \cdot {\text{Electric}}_{{\text{i}}} + \, \beta_{{4}} \cdot {\text{IndustryLeader}}_{{\text{i}}} + \, \beta_{{5}} \cdot {\text{LowEmissions}}_{{\text{i}}} \\ & \quad + \, \beta_{{6}} \cdot {\text{HighEmissions}}_{{\text{i}}} + \, \beta_{{{\text{price}}}} \cdot {\text{Price}}_{{\text{i}}} {\text{U}}\left( {{\text{xi}}} \right) \, = {\text{ Opt}} - {\text{Out}} \\ & \quad + \, \beta_{{1}} \cdot {\text{SignificantIncrease}}_{{\text{i}}} + \, \beta_{{2}} \cdot {\text{LowIncrease}}_{{\text{i}}} + \, \beta_{{3}} \cdot {\text{Electric}}_{{\text{i}}} \\ & \quad + \, \beta_{{4}} \cdot {\text{IndustryLeader}}_{{\text{i}}} + \, \beta_{{5}} \cdot {\text{LowEmissions}}_{{\text{i}}} + \, \beta_{{6}} \cdot {\text{HighEmissions}}_{{\text{i}}} \\ & \quad + \, \beta_{{{\text{price}}}} \cdot {\text{Price}}_{{\text{i}}} \\ \end{aligned}$$ where Opt-Out = dummy for the “none of these/no choice” option; SignificantIncrease = dummy for significant yield increase; LowIncrease = dummy for low yield increase; Electric = dummy for Electric energy; IndustryLeader = dummy for industry leader brand; LowEmissions = dummy variable for CO_2_ low emissions; HighEmissions = dummy variable for CO_2_ high emissions; Price = price in €/equipment. The mentioned β_s_ coefficients can be considered as the marginal utilities of each attribute included in the utility function.

All the coefficients were significant at t+he 95% confidence level (P value), with the exception of the electric power attribute. Positive coefficients attached to leading brand, low emissions and high yields, respectively, suggested preferences for these characteristics, which are expected to increase respondent’s utility. The price attribute coefficient is negative, as expected and postulated by theory, as consumers prefer the less expensive alternative *ceteris paribus*. Similarly, the negative coefficient associated with “High emissions” suggests that respondents on average dislike PA equipment inducing high emission intensities.

To better understand the differences in choice behavior across different respondent segments, a LC approach was used to analyze the data, being it based on the assumption that attributes of the alternatives can be heterogeneous across groups and homogenous within groups. In this study, the LC model allows for categorization of the respondents in segments, which enables useful observations to be made about the study population even though not been able to make statistical inference.

The estimated LC model assumed that respondents could be categorized into three classes, whose unobserved shared characteristics affect choice. In order to identify the optimal number of classes, indicator values were calculated. As detailed in Table 6, the three-class model showed the highest statistical performance, based on indexes such as log-likelihood (LL) function, Akaike information criterion (AIC), Bayesian information criterion (BIC), which was used as a guide to the selection of the optimal model (Ruto and Garrod 2009), and McFadden pseudo R^2^ for different numbers of classes (e.g., Boxal and Adamowicz 2002; Ruto and Garrod 2009; Scarpa and Thiene 2005).

**Table 6:** Latent class models statistics

	LC-2	LC-3	LC-4	LC-5
LL	−3227.07	−3007.96	−2980.53	−2942.65
AIC	2.296	2.147	2.134	2.114
BIC	2.332	2.202	2.208	2.206
HQIC	2.309	2.167	2.161	2.147
McFadden pseudo R^2^	0.18	0.23	0.24	0.25

While the LC model results confirm the abovementioned MNL finding trends, they highlight a differentiated set of preferences among respondents. In fact, each of the three classes was characterized by a unique structure of preferences. The first class is represented by "sustainability seekers". Respondents had 63% probability of membership in this class, which groups low yield and high emission-averse participants who trust leading brands and prefer electric powered equipment and low levels of polluting emissions. They could be considered sustainability supporters since they take into consideration the opportunity to reach good performances both referring to environmental and socio-economic aspects.

The second group includes the "PA best features supporters", with 16% probability of membership. In this class all the variables were statistically significant. The estimates suggest strong positive effects connected to a leading brand. At the same time, electric power, high yields and low emissions increased respondents’ preferences.

The third class grouped the "low emission fans". Respondents had 21% probability of membership in this class. Members of this class appreciated average yield levels, preferred hybrid to electric energy power and demonstrated positive preference for low emissions. This group of buyers is associated with lowest WTP levels compared to the other 2 groups. In addition, in comparison with the first group they mainly take into consideration good performances referred to environmental aspects, while they have settled for ordinary socio-economic aspects (Table 7).

**Table 7:** Main preferences of latent classes

Classes	Preferences mainly toward:
Class 1—Sustainability seekers	Good performances among both socio-economic and environmental aspects
Class 2—PA best features supporters	Leading brand; electric power
Class 3—Low emission fans	Low emissions

The ASC was significant (*P* < 0.05) and positive for class two, but negative for classes one and three, meaning there were preferences toward the ‘none’ option, which could not be explained by the variables contained in our model. In addition, the positive sign for class two suggests that the mean unobserved effects on utility are positive from selecting the “none” option.

Although in a preliminary step we included a number of socio-demographic and behavioral variables, we found that these were not generally significant in explaining the probability of class membership in the latent class model.

The CE permits us to estimate respondents’ willingness to pay (WTP) for each analyzed product attribute and each class. The highest WTP is associated with the presence of a leading brand attribute, precisely class 2 has a willingness to pay € 94,000 for PA equipment provided by an “industry leader” brand. Conversely, the lowest monetary value is associated with the high emission attribute, implying that a € 112,000 discount would be required for Class 2 members to choose the high emission PA setup over the moderate emission option. Respondents are also willing to pay positive monetary amounts for low emission PA investments. With regard to yield performance, lower production results would be preferred to moderate performances only in case of further discounts for all classes (€ 69,000; € 63,000 and € 11,000 respectively).

## Conclusions

According to Akhter and Sofi (2022), PA adoption is able to increase both the quantity and quality of production from the crop fields. Moreover, Mizik (2022) described the economic benefits and potential environmental benefits of PA. In addition, Yin et al. (2021) stated that PA technology improves production by accounting for dynamics within sustainable agricultural systems and ISTAT-CREA (2022) estimated that the introduction of precision farming techniques will result in a reduction in pesticide use estimated at 25 to 40 percentage. However, in spite of several studies demonstrated the importance of PA technology application (Botta et al. 2022; Bouma 2007; Carrer et al. 2022; Colucci et al. 2022), its adoption is still limited in Italy (Finco et al. 2021). A number of studies reported a positive but slow trend (Smart Agrifood Observatory 2021). To fulfill this gap, the Italian National Recovery and Resilience Plan and other complementary funds aim at bringing agricultural innovation to the agricultural sector and ensuring new levers of competitiveness. In addition, also more recent Italian Budget Laws have been trying to support farmers’ investments in PA in order to implement the Guidelines for the development of precision agriculture in Italy adopted by Italian Government during 2017 (Law N° 33,671-22nd December 2017). Moreover, the upcoming 2023-207 Common Agricultural Policy (CAP) aims to strengthen the contribution of agriculture to the EU's environmental and climate objectives providing targeted measures to farms to support transition to practices such as PA. Understanding potential buyers’ needs is therefore essential to increase PA adoption. In this context, our study aimed to increase knowledge of behavior, attitudes and preferences toward PA. Expanding information about PA adoption as a purchase choice could support increasing investments in favor of sustainability. In addition to this, further knowledge about the main obstacles in adopting PA could enhance institutional intervention and favor optimal targeting.

As an exploratory empirical study, the goal of this paper is to develop some initial evidence about the factors influencing PA adoption. Even though potential biases in sample selection may limit the generalization of the results, these could be considered suggestive and provide information for the design of effective policies to induce potential buyers to invest in PA technologies, which can considerably contribute to improve sustainability. Our findings reflect the Italian agricultural industry context, characterized by the presence of several farmers with relatively good knowledge of the potential positive impacts of PA adoption, in particular in terms of social and environmental sustainability. Our study identified the importance of social and professional support in entrepreneurial initiatives to incentivize the adoption of PA, in line with Carli et al. (2017).

Findings gave us the opportunity to discover the main obstacles in PA adoption. In fact, results pointed out that the low propensity to buy is strictly linked to the economic burden of investments and the scarce support from public administrations. These findings are in line with previous studies (e.g., Demeter 2022; Mitchell et al. 2018), who demonstrated that the major barriers to adoption relate to financial aspects. Our results show that high cost is the primary barrier to PA technology adoption. Therefore, any policy reducing this burden through incentives and allowances would positively affect the adoption decision. In a context where input prices are expected to continue to grow due to the general socio-economic trends, a market-based instrument (i.e., an incentive) may be crucial to expand PA adoption. Our analysis also indicates that the provision of professional support is necessary, and even more important of the reputation that industry leaders have. These findings are in line with a number of previous studies (Erickson et al. 2018; Fausti et al. 2018; Kitchen et al. 2002), which observed that the adoption of PA technology and the extraction of its value are both dependent on farmers and their service providers. In detail, Erickson et al. (2018) identified advisers having knowledge about the technologies and the opportunity to have qualified and competent support as crucial in deciding to adopt or not PA technologies.

These evidences suggest that information provided explicitly through a well-known industry label or by consultants can be considered as valid support in solving asymmetric information problems and convincing potential buyers in purchasing PA equipment. In line with the results by Blasch et al. (2021), our study points out the crucial role of both information provision and financial support through incentives to influence the willingness to adopt PA technologies.

The latent class model results provide practical information for both PA producers and retailers to identify market segments and consequently develop marketing strategies to maximize PA awareness and promote wider adoption. The three identified classes show unique profiles and preferences, and their recognition is necessary to fine tune communication contents and channels, also through the involvement of different stakeholders, such as business leaders, government, and sectoral associations. Still, given the shared and widespread concerns over environmental impacts of agricultural activities, industry information campaigns should transversally emphasize the potentially positive role of PA.

Our empirical findings could contribute to the decision-making process of PA industries and service providers strategies and to the formulation of institutional policies aimed at the diffusion of PA.

The generalizability of these results is subject to certain limitations. First of all, given the different structure of the agricultural industry in other countries and regions, a geographical extension of the research is desirable. Moreover, the survey was carried out prior to the COVID-19 pandemic, whose adaptation strategies have radically changed, among other things, also our relationship with technology as individuals and professionally: even in the case of agricultural activities, according to Colussi et al. (2022), digitalization will determine radical industry changes over the upcoming years. However, no studies have analyzed so far the impact of the pandemic on farmers’ preferences toward PA to serve as a comparison with our study, while several authors are studying the potentials of COVID-19 response plans in favor of agricultural digitalization diffusion.

Furthermore, the analysis of actual, rather than potential buyer behavior would support a better and more complete understanding of their preferences. In addition, future studies may replicate our design by extending the model to capture a wider bundle of PA purchase attributes. Despite the limitations of our study, we believe our results add useful information to the existing literature on farmers’ preferences toward PA equipment adoption.

## Data Availability

The datasets used and/or analyzed during the current study are available from the corresponding author on reasonable request.
